# Cannabis Use Among Ugandan Medical Students: Prevalence, Predictors, and Coping Strategies in a Cross‐Sectional Study

**DOI:** 10.1002/brb3.71325

**Published:** 2026-03-29

**Authors:** Ronald Musinguzi, Musa kasujja, Marie Pascaline Sabine Ishimwe, Florent Ishimwe, Maxwell Okello, Theodore Nteziyaremye, Madrine Nakawuki, Ahmed Kiswezi Kazigo, Olabisi Surat Akib, Thomas Dawit, Rogers Kajabwangu, Nzanzu Vivalya Mutume, Umi Omar Bunu, Theoneste Hakizimana

**Affiliations:** ^1^ Department of Psychiatry Kampala International University Ishaka Uganda; ^2^ Department of Obstetrics and Gynecology Kampala International University Ishaka Uganda; ^3^ Department of Pediatrics and Child Health Kampala International University Ishaka Uganda; ^4^ Department of Sciences University of Rwanda Kigali Rwanda; ^5^ Department of Surgery Kampala International University Ishaka Uganda; ^6^ Department of Pathology Kampala International University Ishaka Uganda; ^7^ Department of Public Health Kampala International University Ishaka Uganda; ^8^ Mbarara University of Science and Technology Mbarara Uganda

**Keywords:** cannabis use, coping strategies, cross‐sectional study, CUDIT‐R, DSM‐5‐TR, hazardous use, medical students, Uganda

## Abstract

**Introduction and Aims:**

Cannabis use among university students is a growing concern, particularly in demanding medical programs. We estimated prevalence, identified predictors, and compared coping strategies among medical students in Uganda.

**Design and Methods:**

Cross‐sectional survey of 318 undergraduates at Kampala International University (Western Campus). Cannabis use and hazardous/disordered use were screened with CUDIT‐R (hazardous 8–11; probable use disorder ≥12, DSM‐5‐TR aligned). Coping was measured with the Brief COPE. Predictors were assessed using logistic regression; coping differences with the Wilcoxon rank‐sum test.

**Results:**

Cannabis use prevalence was 30.8% (*n* = 98); **7**.6% met criteria for hazardous use and 9.4% for probable cannabis use disorder. Independent predictors of use were being separated (AOR = 12.00), being single (AOR = 3.45), Catholic faith (AOR = 2.76), and longer time at campus (AOR = 1.16 per year). Users reported higher emotion‐focused and avoidant coping; problem‐focused coping did not differ.

**Discussion and Conclusions:**

Cannabis use among Ugandan medical students is common and associated with relationship status, religion, and time at campus. Coping profiles suggest greater reliance on maladaptive strategies among users. Findings support campus policies and mental‐health programs that integrate substance‐use screening and strengthen adaptive coping skills.

## Graphical abstract

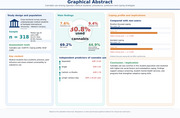

**Abbreviations**
AC: Avoidant coping
AOR: Adjusted odds ratio
CI: Confidence interval
COPE: Coping orientation to problems experienced
COR: Crude odds ratio
CUD: Cannabis use disorder
CUDIT‐R: Cannabis Use Disorder Identification Test–Revised
DSM‐5‐TR: Diagnostic and Statistical Manual of Mental Disorders, Fifth Edition, Text Revision
EFC: Emotion‐focused coping
IQR: Interquartile range
KIU: Kampala International University
KIU‐WC: Kampala International University—Western Campus
PFC: Problem‐focused coping
REC: Research Ethics Committee

## 1 Introduction

Cannabis, derived from the *Cannabis sativa* plant, is the most commonly used illicit psychoactive substance worldwide, with an estimated 219 million users as of 2021, representing approximately 4% of the global population aged 15–64 years (United Nations: Office On Drugs And Crime 2023). Among university students, cannabis use is especially prevalent because of the complex interplay of curiosity, peer influence, stress, perceived safety, and shifting social norms (Degenhardt et al. 2016; Olthuis et al. 2013). Although often perceived as harmless, cannabis use in adolescence and early adulthood has been linked to impaired neurocognitive functioning, academic decline, depression, anxiety, psychosis, and substance dependence (Hall 2015; Silins et al. 2014; Volkow et al. 2014).

Medical students represent a unique population with heightened vulnerability to substance use due to academic pressure, sleep deprivation, and exposure to human suffering (Papazisis et al. 2018; Ayala et al. 2017). A systematic review and meta‐analysis by Papazisis et al. (2018) revealed that approximately one in three medical students worldwide reported lifetime cannabis use, with 8.8% reporting current cannabis use. Regional variations were significant, with higher rates in North America and lower rates in Africa and Asia. However, recent studies suggest increasing cannabis use among African medical students, particularly in urban settings (Adesida et al. 2022; National Authority for the Campaign Against Alcohol and Drug Abuse (NACADA) 2024). This study aimed to determine the prevalence, associated factors, and coping strategies among medical students at a university in Uganda, addressing a significant gap in the literature regarding substance use patterns in East African medical education contexts.

Cannabis use disorder (CUD) is defined by the Diagnostic and Statistical Manual of Mental Disorders, Fifth Edition, Text Revision (DSM‐5‐TR) as a problematic pattern of cannabis use leading to significant impairment or distress, as manifested by at least two of 11 behavioral or physiological criteria within a 12‐month period (American Psychiatric Association 2022). The severity of the disorder is classified as mild (2–3 symptoms), moderate (4–5 symptoms), or severe (6+ symptoms) depending on the number of criteria met. Even in the absence of full CUD, individuals who engage in hazardous use, such as frequent or risky consumption patterns, are at increased risk of developing dependence, experiencing academic or legal problems, and encountering psychiatric comorbidities (Fischer et al. 2017; Hall and Degenhardt 2009).

The relationship between stress, coping mechanisms, and substance use among medical students has gained increasing attention in recent years. Medical education is characterized by high‐stress environments that can trigger maladaptive coping responses (Erschens et al. 2018; Moir et al. 2018). Research suggests that students who use cannabis often do so as a stress‐coping strategy, with significant associations between cannabis use and stress‐related factors (Hyman and Sinha 2009; Moitra et al. 2015). Understanding these relationships is crucial for developing targeted interventions that address both substance use and underlying psychological needs.

The Cannabis Use Disorder Identification Test–Revised (CUDIT‐R) is a widely validated tool used to screen for hazardous and disordered cannabis use, particularly in non‐clinical populations such as students (Adamson et al. 2010). A CUDIT‐R score of 8 or above suggests hazardous use, whereas a score of 12 or more indicates probable cannabis use disorder (Adamson and Sellman 2003). These screening thresholds allow for standardized identification of at‐risk individuals, facilitating early intervention and referral.

Coping strategies the cognitive and behavioral efforts to manage specific external and/or internal demands that are appraised as taxing or exceeding the resources of the person (Lazarus and Folkman 1984) play a crucial role in how students navigate academic stress. The Brief COPE inventory, a validated 28‐item tool, assesses coping across three domains: problem‐focused, emotion‐focused, and avoidant coping (Carver 1997). Problem‐focused coping strategies (e.g., planning, active coping) are considered adaptive, whereas emotion‐focused (e.g., venting, self‐blame) and avoidant strategies (e.g., substance use, denial) are often maladaptive and associated with poor mental health outcomes (Kato 2015; Eisenberg et al. 2012). Understanding the relationship between cannabis uses and coping strategies among medical students is essential for developing effective prevention and intervention programs. This study contributes to this understanding by examining these patterns in a previously understudied population of Ugandan medical students.

## 2 Materials and Methods

### 2.1 Study Design and Setting

We conducted a descriptive, analytical cross‐sectional study in Ishaka, Bushenyi District, and southwestern Uganda. The study was based at Kampala International University—Western Campus (KIU‐WC), a large private medical training institution offering undergraduate programs in medicine, nursing, pharmacy, dental surgery, and allied health sciences. KIU‐WC enrolls **>**5000 students from Uganda and neighboring countries, providing a diverse setting to examine cannabis use and coping among medical trainees.

### 2.2 Participants and Eligibility

The target population comprised all undergraduate students enrolled in medical and health‐related degree programs at KIU‐WC during the 2023–2024 academic year (Bachelor of Medicine and Surgery, Nursing, Pharmacy, Dental Surgery, Radiography, Clinical Medicine and other biomedical sciences).

**Inclusion criteria**: age **≥**18 years, enrolment in an eligible program and electronic informed consent.

**Exclusion criteria**: known physical or psychiatric illness that could impair reliable responses; academic leave or suspension during the study period.

### 2.3 Sample Size

The sample size was calculated via Kish Leslie's formula $n=\frac{Z^{2}PQ}{D^{2}}$(Kish 1965) on the basis of a previous study of cannabis use prevalence in Kenya found to be 25% (Chege et al. 2019), a 5% margin of error, and a 95% confidence level. The minimum sample size obtained was 289 participants. Adding 10% for potential nonresponses yielded a final sample size of 318 participants.

### 2.4 Sampling and Recruitment

We used convenience sampling. Trained class representatives distributed a secure Google Forms link via official WhatsApp and email channels during class breaks. Students completed the anonymous questionnaire electronically. Participation was voluntary and remained open until the target sample size was achieved.

### 2.5 Measures

#### 2.5.1 Sociodemographic and Contextual Variables

Age, sex, year of study, marital status, religion, and duration of university stay were recorded. Additional items covered prior substance use, peer influence, and perceptions of cannabis risks/benefits.

### 2.6 Cannabis Use

Cannabis use was assessed with the Cannabis Use Disorder Identification Test—Revised (CUDIT‐R), an 8‐item screener scored 0–32. We classified hazardous use as 8–11 and probable cannabis use disorder (CUD) as **≥**12 (consistent with DSM‐5‐TR‐aligned cut‐points).

### 2.7 Coping Strategies

Coping was measured using the Brief COPE (28 items; 4‐point Likert: 1 = “I have not been doing this at all” to 4 = “I have been doing this a lot”). Following standard grouping, we derived three domain scores:

- **Problem‐focused** (e.g., active coping, planning, instrumental support)
- **Emotion‐focused** (e.g., emotional support, acceptance, positive reframing)
- **Avoidant** (e.g., denial, substance use, behavioral disengagement, venting)
  Domain scores were calculated by summing relevant items; higher scores indicate greater use of that coping style.

### 2.8 Candidate Predictors

We included sociodemographic covariates and Likert‐style items on peer influence, accessibility, curiosity, stress and perceived legal/health consequences as potential predictors of cannabis use.

### 2.9 Validity and Reliability

Content validity was evaluated by 10 independent experts in substance‐use research who were not part of the study team; items were rated for relevance, with a pre‐specified CVI threshold ≥0.5 considered acceptable. Internal consistency was examined in a pilot (*n* = 10); Cronbach's α > 0.70 for major domains (CUDIT‐R total; Brief COPE domain scores), indicating satisfactory reliability.

### 2.10 Statistical Analysis

Data were exported from Google Forms to Excel, cleaned, and analyzed in Stata 16.0. Categorical variables were summarized as *n* (%). The primary outcome was any cannabis use (yes/no). At the bivariate level, we used logistic regression to screen associations between candidate predictors and cannabis use, reporting crude odds ratios (ORs) with 95% CIs. Variables with theoretical relevance and/or *p* < 0.20 in bivariate models were entered into a multivariable logistic regression to identify independent predictors, reported as adjusted odds ratios (AORs) with 95% CIs and two‐sided *p*‐values. We conducted basic model diagnostics (e.g., multicollinearity checks and **s**pecification tests). Because coping domain scores were non‐normally distributed, we compared users versus non‐users with the Wilcoxon rank‐sum test. Statistical significance was set at *p* < 0.05.

### 2.11 Ethics

Ethical approval was obtained from the Kampala International University Research Ethics Committee (KIU‐REC‐2023‐0124). Participants provided electronic informed consent before accessing the survey. Responses were anonymous, confidentiality was maintained throughout, and participants could withdraw at any time without penalty.

## 3 Results

### 3.1 Participant Characteristics

A total of 516 undergraduate medical students were invited to participate. Of these, 318 completed the survey, resulting in a response rate of 61.6%. All the participants provided informed consent and submitted fully completed responses, yielding no missing data.

The median age of the participants was 25 years (IQR: 23–27). The majority were male (67.9%), single (83.0%), and Ugandan nationals (71.1%). Most students were enrolled in clinical medicine and dentistry (42.1%) or pharmacy (18.6%) programs. The full distribution of participant characteristics is presented in Table 1.

**TABLE 1:** Descriptive statistics of undergraduate students at KIU‐Western Campus (*N* = 318).

Study variables	Total (*N* = 318) *n* (%)
**Age (years), median (IQR)**	25 (23, 27)
**Sex**	
Female	102 (32.1%)
Male	216 (67.9%)
**Religion**	
Muslim	46 (14.5%)
Catholic	156 (49.1%)
Born again	59 (18.6%)
Protestant	57 (17.9%)
**Marital status**	
Married	36 (11.3%)
Separated	18 (5.7%)
Single	264 (83.0%)
**Nationality**	
Ugandan	226 (71.1%)
Foreigner	92 (28.9%)
**School/faculty**	
Allied health	41 (12.9%)
Biomedicals	35 (11.0%)
Clinical medicine & dentistry	134 (42.1%)
Nursing	48 (15.1%)
Pharmacy	59 (18.6%)
Science & technology	1 (0.3%)
**Duration at campus (years), median (IQR)**	2.5 (1.5, 5.5)
**Family history of mental illness**	
No	262 (82.4%)
Yes	56 (17.6%)
**Chronic medical condition**	
No	299 (94.0%)
Yes	19 (6.0%)
**Sponsorship type**	
Government	44 (13.8%)
KIU scholar	30 (9.4%)
Private	244 (76.8%)

IQR, interquartile range; KIU, Kampala International University.

### 3.2 Prevalence of Cannabis Use Among Undergraduate Students at KIU, Western Campus

**FIGURE 1:**
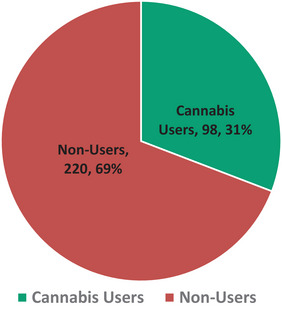
A pie chart showing the prevalence of cannabis use.

Among the 318 participants, 98 (30.8%) reported cannabis use, whereas 220 (69.2%) indicated no cannabis use (Figure 1). Among the 98 cannabis users, 44 (44.9%) did not meet the CUD criteria, 30 (30.6%) met the criteria for CUD, and 24 (24.5%) reported hazardous use.

Thus, nearly half of the participants used cannabis without meeting clinical or hazardous thresholds, whereas the remainder exhibited problematic patterns. This distribution emphasizes the importance of targeted screening and interventions within this subgroup of cannabis users.

### 3.3 Factors Associated With Cannabis Use

Marital status, religion, and duration at campus were independent predictors of cannabis use. Separated students were 12 times more likely, and single students were 3.45 times more likely to use cannabis than married students are. Catholics had 2.76 times greater odds of cannabis use than Muslims did. Each additional year at campus increased the odds of cannabis use by 16% (Table 2).

**TABLE 2:** Predictors of cannabis use.

Study variables	Cannabis use		Bivariate analysis		Multivariate analysis	
	No (*n* = 220)	Yes (*n* = 98)	COR (95% CI)	*p*‐value	AOR (95% CI)	*p*‐value
**Age (years), median IQR**	25 (23, 27)	25 (24, 26)	0.97 (0.90–1.03)	0.296	0.95 (0.87–1.03)	0.218
**Sex**						
Female	76 (34.5%)	26 (26.5%)	Ref	—	Ref	—
Male	144(65.5%)	72 (73.5%)	1.46 (0.86–2.48)	0.159	1.68 (0.92–3.06)	0.093
**Religion**						
Muslim (reference)	35 (15.9%)	11 (11.2%)	Ref	—	Ref	—
Catholic	96 (43.6%)	60 (61.2%)	1.99 (0.94–4.21)	0.073	2.76 (1.22–6.24)	**0.015**
Born again	47 (21.4%)	12 (12.2%)	0.81 (0.32–2.05)	0.661	0.97 (0.35–2.64)	0.947
Protestant	42 (19.1%)	15 (15.3%)	1.14 (0.46–2.79)	0.780	1.32 (0.51–3.40)	0.567
**Marital status**						
Married (reference)	31 (14.1%)	5 (5.1%)	Ref	—	Ref	—
Separated	10 (4.5%)	8 (8.2%)	4.96 (1.32–18.67)	0.018	12.00 (2.66–54.08)	**0.001**
Single	179(81.4%)	85 (86.7%)	2.94 (1.11–7.84)	0.031	3.45 (1.13–10.54)	**0.030**
**Nationality**						
Foreign (reference)	63 (28.6%)	29 (29.6%)	Ref	—	Ref	—
Ugandan	157(71.4%)	69 (70.4%)	0.95 (0.57–1.61)	0.862	—	—
**Faculty**						
Allied health (reference)	30 (13.6%)	11 (11.2%)	Ref	—	Ref	—
Biomedicals	27 (12.3%)	8 (8.2%)	0.81 (0.28–2.31)	0.690	0.68 (0.22–2.06)	0.491
Clinical medicine & dentistry	83 (37.7%)	51 (52.0%)	1.68 (0.77–3.63)	0.191	1.22 (0.52–2.87)	0.653
Nursing	33 (15.0%)	15 (15.3%)	1.24 (0.49–3.12)	0.648	1.04 (0.38–2.86)	0.936
Pharmacy	46 (20.9%)	13 (13.3%)	0.77 (0.31–1.94)	0.581	0.63 (0.23–1.69)	0.356
**Duration at campus (years)**	1 (0.5%)	0 (0.0%)	1.10 (0.98–1.23)	0.104	1.16 (1.02–1.33)	**0.027**
**Family history of mental illness**	2.5 (1.5, 5.5)	2.5 (1.5, 5.5)				
No	185(84.1%)	77 (78.6%)	Ref	—	Ref	—
Yes	35 (15.9%)	21 (21.4%)	1.44 (0.79–2.63)	0.234	—	—
**Chronic medical condition**						
No	208 (94.5%)	91 (92.9%)	Ref	—	Ref	—
Yes	12 (5.5%)	7 (7.1%)	1.33 (0.51–3.50)	0.559	1.20 (0.39–3.66)	0.748
**Scholarship**						
Government	26 (11.8%)	18 (18.4%)	Ref	—	Ref	—
KIU scholarship	25 (11.4%)	5 (5.1%)	0.29 (0.09–0.90)	0.032	0.30 (0.09–1.05)	0.059
Private	169(76.8%)	75 (76.5%)	0.64 (0.33–1.24)	0.186	0.69 (0.33–1.44)	0.322

Ref, reference category; AOR, adjusted odds ratio; COR, crude odds ratio.

*Note*: Bolded *p*‐values indicate statistically significant (<0.05).

Table 3 highlights students' knowledge, attitudes, and perceptions, with peer influence (68.9%), curiosity (73.3%), and academic stress (74.8%) as key drivers of cannabis use. Prior alcohol/tobacco use (77.7%) was widely recognized as a risk factor.

**TABLE 3:** Students’ knowledge, attitudes, and perceptions regarding cannabis use (*N* = 318).

Knowledge, attitudes, and perceptions	Level	*n* (%)
**Peer influence plays a significant role in cannabis use among undergraduate students**		
	Agree	219 (68.87%)
	Neutral	50 (15.72%)
	Disagree	49 (15.41%)
**Curiosity‐driven experimentation is a common reason for cannabis initiation**		
	Agree	233 (73.27%)
	Neutral	59 (18.55%)
	Disagree	26 (8.18%)
**Some students perceive cannabis use as safe and non‐addictive, leading to increased experimentation**		
	Agree	225 (70.75%)
	Neutral	64 (20.13%)
	Disagree	29 (9.12%)
**Positive attitudes toward cannabis, such as perceiving it as a harmless recreational substance, are associated with its use**		
	Agree	238 (74.84%)
	Neutral	52 (16.35%)
	Disagree	28 (8.81%)
**Cultural and social norms influence the prevalence of cannabis use**		
	Agree	179 (74.30%)
	Neutral	23 (9.50%)
	Disagree	39 (16.20%)
**Some students believe that cannabis use helps cope with academic stress and enhances concentration**		
	Agree	238 (74.84%)
	Neutral	46 (14.47%)
	Disagree	34 (10.69%)
**Legalization and policy context impact cannabis use**		
	Agree	222 (69.81%)
	Neutral	50 (15.72%)
	Disagree	46 (14.47%)
**Mental health issues are associated with cannabis use**		
	Agree	231 (72.64%)
	Neutral	60 (18.87%)
	Disagree	27 (8.49%)
**Prior use of alcohol and tobacco is linked to cannabis use**		
	Agree	247 (77.67%)
	Neutral	44 (13.84%)
	Disagree	27 (8.49%)

### 3.4 Coping Mechanisms Among Cannabis Users and Nonusers

The study assessed coping mechanisms among cannabis users and nonusers, focusing on problem‐focused coping, emotion‐focused coping, and avoidant coping. The Wilcoxon rank‐sum test was used to compare the median scores between the two groups (Table 4).

**TABLE 4:** Comparison of coping mechanisms between cannabis users and non‐users (*N* = 318).

Coping mechanism	Median (IQR)	No cannabis use (*n* = 220)	Cannabis use (*n* = 98)	z‐score	*p*‐value
**Problem‐focused coping (PFC)**	17 (13, 20)	17 (13, 20)	17 (13, 20)	−1.913	0.056
**Emotion‐focused coping (EFC)**	24 (18, 29)	24 (18, 29)	25 (19, 30)	−2.689	0.007**
**Avoidant coping (AC)**	14.5 (10, 19)	14 (10, 18)	16 (12, 21)	−4.319	<0.001**

**Significance level: *p* < 0.05.

Compared with nonusers, cannabis users reported significantly higher scores for emotion‐focused coping (median 22 vs. 20, *p* = 0.007) and avoidant coping (median 24 vs. 19, *p* < 0.001). No significant difference was observed in problem‐focused coping (median 21 vs. 20, *p* = 0.056), although the trend suggested slightly greater use of problem‐focused strategies among cannabis users.

*Note*: The higher scores for emotion‐focused and avoidant coping among cannabis users suggest that these students may use cannabis as part of a broader pattern of maladaptive stress management.

## 4 Discussion

This study revealed that 30.8% of undergraduate medical students at the KIU–Western Campus reported cannabis use, with 17.0% meeting the criteria for hazardous or disordered cannabis use.

These figures reflect a rising concern within academic institutions, particularly in low‐resource settings where psychosocial support systems may be limited.

The prevalence observed in our study is comparable to the global estimate of 31.4% lifetime cannabis use among medical students reported by Papazisis et al. (2018) in their systematic review and meta‐analysis. However, these rates are higher than those in previous reports from East African medical schools, which have typically reported prevalence rates of 2%–14% (Chege et al. 2019; Kurui and Ogoncho 2019). This discrepancy may reflect regional variations, methodological differences, or a genuine increase in cannabis use among medical students in Uganda. The high proportion of students demonstrating hazardous use or probable cannabis use disorder (CUD) underscores a pattern observed globally university students, particularly in high‐pressure academic environments, are vulnerable to problematic substance use (Volkow et al. 2014; Bonn‐Miller et al. 2007). Hazardous use among students has been linked to poor academic performance, mental health challenges, and increased risk of dependency (Holm et al. 2010).

Research by Mader et al. (2016) suggested that medical students may be at particular risk due to the combination of high academic demands, clinical stressors, and limited time for healthy stress management. The transition from preclinical to clinical years has been identified as a particularly vulnerable period, with increased substance use reported as students adjust to patient care responsibilities (Ayala et al. 2017).

Marital status, religion, and duration at university were significantly associated with cannabis use. Compared with their married peers, separated students had 12‐fold greater odds, whereas single students had 3.45‐fold greater odds of cannabis use. This finding is consistent with the literature showing that marital stability offers protective social and emotional support, reducing the odds of maladaptive coping (Adesida et al. 2022; Dachew et al. 2015). Conversely, separation is often associated with distress and increased substance use (Olashore et al. 2018).

Religious affiliation also emerged as a significant predictor, with Catholic students being a total of 2.76 times more likely to use cannabis than Muslims are. This finding supports earlier research indicating that Islamic teachings often more restrictive regarding drug use may offer stronger behavioral regulation (Kurui and Ogoncho 2019; Okafor et al. 2023). Similar patterns have been observed in studies from Nigeria and Ethiopia, where religious prohibition was associated with lower substance use rates (Adesida et al. 2022; Kurui and Ogoncho 2019).

The odds of cannabis use increased by 16% with each additional year spent at university, likely due to cumulative exposure to peer influence, stress, and substance access (National Authority for the Campaign Against Alcohol and Drug Abuse (NACADA) 2024; Okafor et al. 2023). These results align with South African data showing higher cannabis use in final‐year students (Inaç et al. 2021). Longitudinal studies have demonstrated that substance use patterns often escalate throughout university education, with particular vulnerability during transition periods and high‐stress academic cycles (Skidmore et al. 2016).

Although gender, mental health history, and financial background were not significantly associated with use in this sample, global evidence suggests that male students and those with mental health vulnerabilities are at increased risk (Volkow et al. 2014; Adesida et al. 2022). The lack of statistical significance in this study may reflect sample distribution or underreporting due to stigma.

Perceived social and psychological drivers were prominent. The majority of the students cited curiosity (73.3%), academic stress (74.8%), and peer influence (68.9%) as key motivations for cannabis initiation. These findings align with findings from Nigeria, Botswana, and Ethiopia, where students often experiment due to peer modeling and curiosity about psychoactive effects (Kurui and Ogoncho 2019; Olashore et al. 2018).

A concerning proportion (70.8%) viewed cannabis as safe or non‐addictive, highlighting a gap in awareness. Similar misconceptions have been linked to increased experimentation in Tanzania and Egypt (Mavura et al. 2022; Shalaby and Soliman 2019). This perception gap may be particularly problematic among medical students, who will eventually counsel patients on substance use risks (Hoffmann and Weber 2010).

The high endorsement of academic stress as a motivator for cannabis use (74.8%) is particularly noteworthy and is consistent with the literature on substance use as a stress‐coping mechanism (Hyman and Sinha 2009). Medical education is characterized by high‐pressure environments, with students facing academic demands, clinical responsibilities, and often sleep deprivation (Erschens et al. 2018). Without adequate institutional support and healthy coping alternatives, students may turn to substances for temporary relief (Moir et al. 2018).

Our findings revealed significant differences in coping profiles between cannabis users and nonusers. Cannabis users demonstrated greater reliance on emotion‐focused and avoidant coping strategies, which are generally considered maladaptive (Kato 2015). This pattern is consistent with the self‐medication hypothesis, which suggests that individuals use substances to manage negative emotional states (Khantzian 1997).

The higher scores for avoidant coping among cannabis users (median 24 vs. 19, *p* < 0.001) align with previous research showing associations between avoidance‐oriented coping and substance use (Hyman and Sinha 2009; Moitra et al. 2015). Avoidant strategies, including substance use itself, denial, and behavioral disengagement, may provide temporary relief but ultimately exacerbate stress and psychological distress (Kato 2015).

Interestingly, cannabis users also showed slightly elevated emotion‐focused coping (median 22 vs. 20, *p* = 0.007). While some emotion‐focused strategies, such as seeking emotional support, can be adaptive, others, such as self‐blame and rumination, are linked to poorer outcomes (Eisenberg et al. 2012). The specific components driving this difference warrant further investigation.

The lack of a significant difference in problem‐focused coping (*p* = 0.056) suggests that cannabis users may not necessarily lack adaptive coping skills but rather may supplement these with maladaptive strategies. This nuanced understanding is important for intervention design, as it suggests that building on existing problem‐solving capabilities while reducing reliance on avoidant strategies may be effective (Bravo et al. 2016).

Recent research by Mader et al. (2016) and Erschens et al. (2018) emphasized the importance of institutional approaches to stress management in medical education. Evidence‐based interventions, including mindfulness training, cognitive‒behavioral techniques, and peer support programs, have shown promise in reducing both stress and substance use among medical students (Moir et al. 2018; Dyrbye et al. 2017).

### 4.1 Strengths and Limitations

This study has several strengths, including the use of validated assessment tools (CUDIT‐R and Brief COPE), and a relatively large sample size. The anonymous online format likely reduced social desirability bias in reporting sensitive behaviors.

However, limitations should be acknowledged. The cross‐sectional design precludes causal inferences about the relationship between coping strategies and cannabis use. The convenience sampling approach may limit generalizability, and self‐reported data are subject to recall bias. Additionally, the study was conducted at a single institution, and patterns may differ at other universities or in different regions of Uganda. In addition, the survey response rate was moderate, and students who did not participate may have differed systematically from respondents, introducing potential nonresponse or self‐selection bias. As a result, the prevalence of cannabis use and the strength of its associations with related factors may have been either under‐ or over‐estimated

## 5 Conclusion

Cannabis use was common among medical students at this Ugandan university, and a notable subgroup screened positive for risky use patterns consistent with hazardous use or probable cannabis use disorder. Relationship status, religious affiliation, and longer duration at campus emerged as significant predictors, suggesting that social context and cumulative exposure within the university environment may shape vulnerability. Students who used cannabis reported greater reliance on emotion‐focused and avoidant coping strategies, indicating that cannabis use may coexist with maladaptive stress‐management patterns. These findings support strengthening campus‐based prevention and screening, integrating mental health services that promote adaptive coping, and prioritizing targeted support for higher‐risk student groups, while future longitudinal studies should clarify temporal relationships between stress, coping trajectories, and cannabis use.

## Recommendations

These findings have important implications for medical education and student support services. First, they highlight the need for routine screening for substance use among medical students, particularly those with identified risk factors. Second, they suggest that interventions should address both substance use behaviors and underlying coping deficits. Third, they underscore the importance of institutional approaches to stress management, including curriculum modifications to reduce unnecessary stressors and the provision of accessible mental health services.

Future research should explore longitudinal patterns of cannabis use and coping throughout medical education, examine protective factors that promote resilience, and evaluate the effectiveness of targeted interventions. Additionally, qualitative studies could provide deeper insights into students' motivations and experiences with cannabis use in the context of medical education.

Medical schools are responsible not only for training competent clinicians but also for supporting students' well‐being throughout their educational journeys. Addressing cannabis use and promoting healthy coping strategies are essential components of this responsibility, with potential benefits for both student welfare and future patient care.

## Author Contributions

RM and TH conceived the study, designed the methodology, and drafted the manuscript. MK, MPSI, and TH contributed to the data analysis and interpretation. FI, NT, AKK, UOB, OSA, TH, MO, MN, TD, NVM, and JI participated in the data collection and literature review. All the authors read and approved the final manuscript.

## Funding

The authors have nothing to report.

## Ethics Statement

This study was approved by the Kampala International University Research Ethics Committee (KIU‐REC‐2023‐0124). All participants provided electronic informed consent before participating in the study.

## Conflicts of Interest

The authors declare that they have no competing interests.

## Data Availability

The datasets used and analyzed during the current study are available from the corresponding author upon reasonable request.
